# Clinical Outcomes in Patients with Ischemic versus Non-Ischemic Cardiomyopathy after Angiotensin-Neprilysin Inhibition Therapy

**DOI:** 10.3390/jcm10214989

**Published:** 2021-10-27

**Authors:** Mohammad Abumayyaleh, Christina Pilsinger, Ibrahim El-Battrawy, Marvin Kummer, Jürgen Kuschyk, Martin Borggrefe, Andreas Mügge, Assem Aweimer, Ibrahim Akin

**Affiliations:** 1 First Department of Medicine, University Medical Center Mannheim, University Heidelberg, 68167 Mannheim, Germany; mohammad.abumayyaleh@medma.uni-heidelberg.de (M.A.); christina.pilsinger@gmail.com (C.P.); marvin.kummer@yahoo.de (M.K.); Juergen.kuschyk@medma.uni-heidelberg.de (J.K.); Martin.borggrefe2006@gmail.com (M.B.); Ibrahim.Akin@umm.de (I.A.); 2 DZHK (German Centre for Cardiovascular Research) Partner Site, Heidelberg-Mannheim, 68167 Mannheim, Germany; 3 Department of Cardiology and Angiology, Bergmannsheil University Hospitals, Ruhr University of Bochum, 44789 Bochum, Germany; andreas.muegge@bergmannsheil.de (A.M.); assem.aweimer@bergmannsheil.de (A.A.)

**Keywords:** ARNI, ICMP, NICMP, sacubitril/valsartan, tachyarrhythmias, outcomes

## Abstract

Background: The angiotensin receptor-neprilysin inhibitor (ARNI) decreases cardiovascular mortality in patients with chronic heart failure with a reduced ejection fraction (HFrEF). Data regarding the impact of ARNI on the outcome in HFrEF patients according to heart failure etiology are limited. Methods and results: One hundred twenty-one consecutive patients with HFrEF from the years 2016 to 2017 were included at the Medical Centre Mannheim Heidelberg University and treated with ARNI according to the current guidelines. Left ventricular ejection fraction (LVEF) was numerically improved during the treatment with ARNI in both patient groups, that with ischemic cardiomyopathy (*n* = 61) (ICMP), and that with non-ischemic cardiomyopathy (*n* = 60) (NICMP); *p* = 0.25. Consistent with this data, the NT-proBNP decreased in both groups, more commonly in the NICMP patient group. In addition, the glomerular filtration rate (GFR) and creatinine changed before and after the treatment with ARNI in both groups. In a one-year follow-up, the rate of ventricular tachyarrhythmias (ventricular tachycardia and ventricular fibrillation) tended to be higher in the ICMP group compared with the NICMP group (ICMP 38.71% vs. NICMP 17.24%; *p* = 0.07). The rate of one-year all-cause mortality was similar in both groups (ICMP 6.5% vs. NICMP 6.6%; log-rank = 0.9947). Conclusions: This study shows that, although the treatment with ARNI improves the LVEF in ICMP and NICMP patients, the risk of ventricular tachyarrhythmias remains higher in ICMP patients in comparison with NICMP patients. Renal function is improved in the NICMP group after the treatment. Long-term mortality is similar over a one-year follow-up.

## 1. Introduction 

The angiotensin receptor-neprilysin inhibitor (ARNI), which consists of the neprilysin inhibitor sacubitril and the angiotensin receptor blocker (ARB) valsartan, is used to treat symptomatic heart failure with a reduced ejection fraction (HFrEF) [1]. Neprilysin, as an endopeptidase, degrades and disables natriuretic peptides (NP) [2]. Valsartan binds to angiotensin type I receptor (AT1) and blocks angiotensin II. The combined effect of ARNI is associated with better hemodynamics in patients with heart failure (HF) compared with ARB alone [3]. In PARADIGM-HF (Prospective Comparison of ARNI with ACEI to Determine Impact on Global Mortality and Morbidity in Heart Failure), treatment with ARNI in patients suffering from HFrEF was associated with a lower rate of cardiovascular deaths as well as hospitalization for HF compared with enalapril [1]. In patients with heart failure with preserved ejection fraction (HFpEF), ARNI did not reduce the total hospitalization rate due to HF and deaths from cardiovascular causes [4]. On the other hand, the improvement in glycemic control in patients suffering from HFrEF and type 2 diabetes was observed [5]. In addition, a patient under ARNI therapy had an improved health status compared with patients without ARNI therapy and with decreased heart failure symptoms, improved physical functions, and better quality of life [6]. In one analysis of PARADIGM-HF, the advantage of ARNI over angiotensin-converting enzyme inhibitor (ACEI) did not depend on a certain etiology [7]. However, ischemic etiology was an independent predictor for discontinuation of the treatment with ARNI [8]. Further data on the role of etiology in the treatment of ARNI are limited.

Regarding the treatment with ACEI or ARB, one study has shown that ACEI or ARB was associated with a lower survival rate in ischemic cardiomyopathy (ICMP) compared with non-ischemic cardiomyopathy (NICMP) [9]. At long-term follow-up, another study presented a higher mortality rate in patients suffering from ICMP than in patients suffering from NICMP who were medicated with ACEI or ARB [10]. Furthermore, atrial fibrillation (AF) patients with ICMP showed a higher rate of cardiovascular death in comparison with AF patients with NICMP, under treatment with ACEI or ARB [11]. 

Since there was a lack in the data concerning ARNI therapy with respect to different etiologies of HF, we analyzed a consecutive patient cohort with ICMP versus NICMP to explore the impact of ARNI in patients with different HF etiologies. The present study aims to compare the one-year mortality in HFrEF patients with ICMP compared with HFrEF patients with NICMP after the ARNI treatment.

## 2. Methods

**Figure 1:**
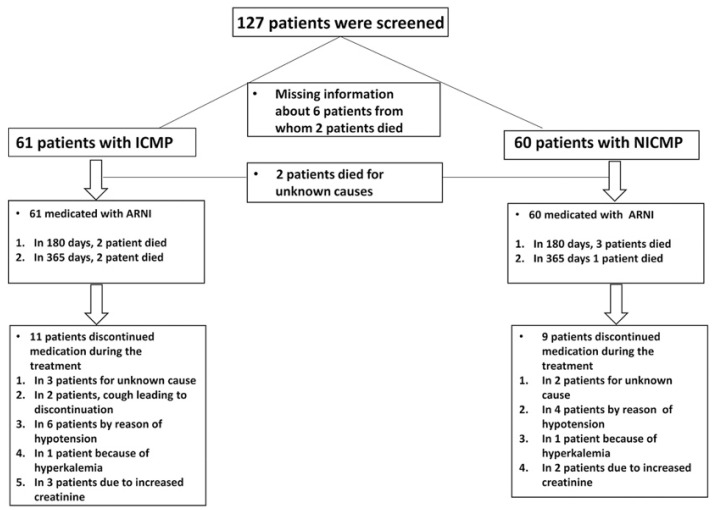
Study design.

One hundred twenty-seven consecutive patients diagnosed with HFrEF between 2016 and 2017 at the University Medical Centre Mannheim Heidelberg University were initially screened. Six patients were excluded because of uncertain information regarding the etiology of HF. However, the data of one hundred twenty-one patients were complete (Figure 1). Chronic HFrEF was diagnosed in accordance with the HF guidelines of the European Society of Cardiology [12]. Patients were included if they (1) had HF symptoms with New York Heart Association (NYHA) functional class II to IV despite optimal HF medication, (2) hd a left ventricular ejection fraction (LVEF) ≤40%, and (3) tolerated ARNI therapy (initially at a dose of 24/26 mg twice daily, which was increased to 97/103 mg twice daily). 

One hundred twenty-one patients were divided with respect to HF etiology into two groups: ICMP (*n* = 61) and NICMP (*n* = 60). The data about medication intake and side effects as well as clinical outcomes were collected by chart review and/or telephone review. Treatment was discontinued in patients who suffered side effects (cough, symptomatic hypotension, hyperkaliemia, increased creatinine, and depression of kidney function). Clinical parameters (systolic and diastolic blood pressure as well as heart rate), laboratory values (glomerular filtration rate (GFR), creatinine, potassium, and N-terminal prohormone of brain natriuretic peptide (NT-proBNP)), electrocardiogram (ECG) data, and medical history were collected before and after the treatment with ARNI at six- and twelve-month follow-ups. Furthermore, echocardiography was conducted before ARNI treatment and during clinical visits at six- and twelve-month follow-ups after the beginning of the treatment. The presentation of ventricular tachyarrhythmias was assessed by interrogating implantable cardioverter-defibrillators (ICD) or cardiac resynchronization therapy devices (CRT). 

Worsening renal function (WRF) was defined as a change in serum creatinine, specifically as an increase in serum creatinine >0.3 mg/dL compared with baseline creatinine value or an increase of serum creatinine within seven days [13,14]. Estimated GFR was calculated by the abbreviated MDRD equation.

This study was conducted in accordance with the Declaration of Helsinki. The study protocol was recently approved by the Ethics Committee of the University Medical Centre Mannheim.

### 2.1. Outcome 

We described one-year all-cause mortality as the primary endpoint. Ventricular tachyarrhythmias, change in kidney function, and the improvement of LVEF as secondary endpoints were also evaluated. 

### 2.2. Statistical Analysis 

Continuous variables with a normal distribution are presented as mean ± standard deviation, and those with a non-normal distribution are presented as median (min-max). Categorical variables are presented as frequencies and percentages (%). The Shapiro–Wilk test was used to test normal distribution. Student’s *t*-test and Mann–Whitney U test were used to compare normal or nonnormal distributions of continuous variables, respectively. The Chi-squared test or Fischer’s exact test was used for distribution analysis to compare categorical variables. Wilcoxon’s signed-rank test was used for paired nonparametric quantitative variables, while the McNemar test was used for paired qualitative variables. We estimated the survival rate using the Kaplan–Meier estimation. Predictors of mortality were identified by univariate analysis. Predictors with *p* < 0.05 were analyzed by the Cox multivariate regression. Statistical analysis was performed with SPSS, Version 23.0 (IBM SPSS Statistics for Windows. Armonk, NY, USA). *p* < 0.05 was recognized as statistically significant. 

## 3. Results

### 3.1. Baseline Characteristics before and after Sacubitril-Valsartan

The patient characteristics before and after ARNI are listed in Table 1. One hundred twenty-seven consecutive patients were screened between the years 2016 and 2017. Six patients were excluded because of a lack of information about HF etiology. One hundred twenty-one patients were divided with respect to HF etiology into two groups: ICMP (*n* = 61) and NICMP (*n* = 60). Forty-four patients (73.3%) were documented to have an idiopathic dilated cardiomyopathy, five patients (8.3%) had hypertrophic cardiomyopathy, four patients (6.7%) had a mixed phenotype (dilated and hypertrophic), one patient (1.7%) had non-compaction cardiomyopathy, one patient (1.7%) had chemotherapy-related cardiomyopathy, and five patients (8.3%) had other types of cardiomyopathy. The risk factors for cardiovascular disease in both groups were comparable (smoking: 24.07% in ICMP vs. 25.45% in NICMP, *p* = 0.87; diabetes mellitus type II: 39.34% in ICMP vs. 27.59% in NICMP, *p* = 0.18; and hypertension: 76.67% in ICMP vs. 62.96% in NICMP, *p* = 0.11). GFR and creatinine changed before and after the treatment with ARNI in both groups: GFR from 52.91 ± 26.02 mL/min to 42.10 ± 19.05 mL/min and creatinine from 1.60 ± 0.52 mg/dL to 1.99 ± 1.24 mg/dL in ICMP patients, *p* = 002 and *p* = 0.01, and GFR from 60.63 ± 21.43 mL/min to 70.52 ± 28.08 mL/min and creatinine from 1.19 ± 0.53 mg/dL to 1.19 ± 0.47 mg/dL in NICMP patients, *p* = 0.001 and *p* = 0.26. In contrast, NT-proBNP decreased significantly after the treatment with ARNI in both groups (in the ICMP group, from 6266.68 ng/L to 6191.76 ng/L, *p* = 0.24, and in the NICMP group, from 5132.82 ng/L to 1170.69 ng/L, *p* = 0.01. In addition, systolic and diastolic blood pressure (BP) decreased in both groups (in systolic BP in the ICMP group, from 132.14 ± 30.28 mmHg to 118.62 ± 27.17 mmHg, *p* = 0.28, and in the NICMP group, from 128.73 ± 14.50 to 113.81 ± 17.67 mmHg, *p* = 0.23). Furthermore, EF was numerically improved at follow-up in both groups (Table 1).

### 3.2. Side Effects Leading to Treatment Discontinuation

#### 3.2.1. ICMP Group 

Collectively, eleven ICMP patients (18%) discontinued the medication during ARNI therapy. Three patients were for an unknown cause. Two patients developed a cough that led them to stop the treatment. In six patients, symptomatic hypotension was documented. One patient had hyperkaliemia, and three other patients developed a renal impairment and a clinically relevant increase in creatinine. In seven cases, the patients suffered two side effects at the same time (Figure 1). 

#### 3.2.2. NICMP Group 

Nine NICMP patients (15%) discontinued ARNI treatment, in two cases, for an unknown cause. Four patients had symptomatic hypotension. One patient had hyperkaliemia, and two patients developed WRF (Figure 1). 

### 3.3. Incidence of Ventricular Tachyarrhythmia and Mortality in ICMP and NICMP Patients after ARNI

**Figure 2:**
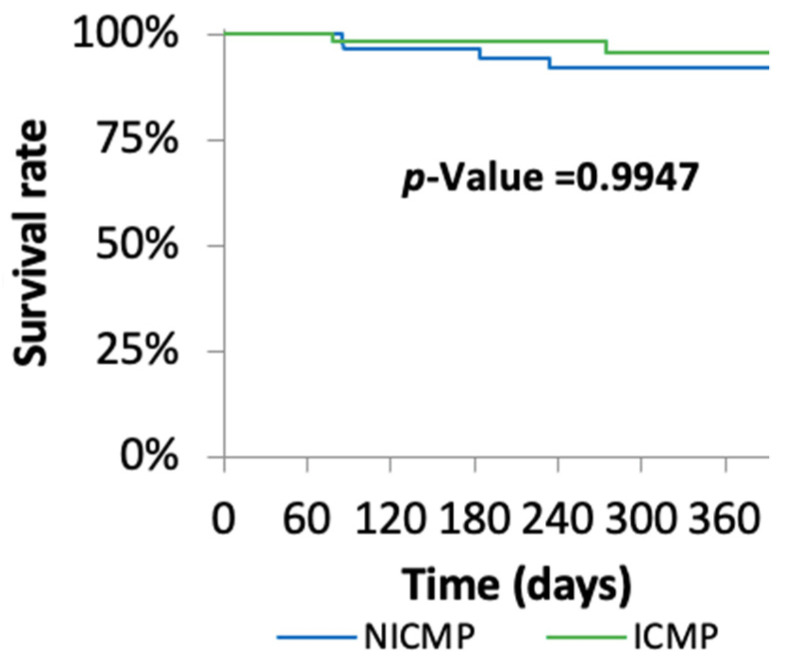
Kaplan–Meier survival analysis.

At one-year follow-up, ventricular tachyarrhythmias including ventricular fibrillation (VF), non-sustained ventricular tachycardia (nsVT), and ventricular tachycardia (VT) were lower in the NICMP group compared with the ICMP group (17.24% vs. 38.71%; *p* = 0.07) (Table 2). However, the long-term mortality rate was similar in both groups (Figure 2).

### 3.4. The Impact of Device Therapy on Outcome and Predictors for Mortality

At baseline, CRT-D and ICD were implanted in 24.59% and 68.85% of ICMP patients and in 32.2% and 46.67% of NICMP patients, respectively. The impact of device therapy on outcome was also evaluated. In this sub-analysis, the mortality rate in ICD patients was lower compared with that in patients with a CRT-D (6.1% vs. 10%; *p* < 0.001). Concerning ventricular tachyarrhythmias, the rate of documented events in ICD patients was significantly higher than in CRT-D patients (45.1% vs. 32%; *p* = 0.003) (Table 3). In the multivariate analysis, the aldosterone antagonist was determined as a relevant predictor for the reduction in mortality (HR 0.21; 95%CI 0.05–0.82; *p* = 0.03, Table 4).

## 4. Discussion 

The current study presents the incidence of ventricular tachyarrhythmias and one-year mortality in patients with ICMP in comparison with NICMP patients after ARNI. The main findings of this study are as follows: (1) The one-year mortality was similar in both groups. (2) Although ventricular tachyarrhythmias (VF, nsVT, and VT) were lower in NICMP compared with the ICMP group at one-year follow-up, LVEF increased in both groups. (3) The ICMP group suffered impairments of kidney function compared with the NICMP group. (4) Aldosterone antagonists were determined as a predictor for the reduction in mortality.

Angiotensin II receptor and neprilysin inhibition were more effective in reducing the risk for cardiovascular death or hospitalization in HF patients in comparison with ACEI alone. In addition, the PARADIGM-HF trial supported the switch from ACEI or ARB to ARNI in the treatment of symptomatic chronic heart failure [1]. Generally, the mortality rate in ICMP patients might be higher than in NICMP patients [15,16,17]. Furthermore, the data have shown that NICMP patients might suffer from lower events of ventricular tachyarrhythmias and do not require an ICD implantation at an advanced age [17]. 

In the present study, the one-year all-cause mortality rate was identical in both groups (ICMP, 6.5% vs. NICMP, 6.6%). Balmforth et al. reported that ARNI had a benefit in all patients regardless of the HF etiology. However, the mortality rate was higher in patients that suffered from ICMP compared with NICMP [7]. The Multicenter Defibrillator Implantation Trial (MADIT-II-trial) presented a high mortality in ICMP of 19.8% at the twenty-month follow-up. Considering the longer follow-up time than our study, this rate is higher than the rate in our study [18]. In addition, it was observed that one-year mortality rate was 29.5% after ARNI in patients, 65.2% from whom suffered from ICMP [19]. In the DEFINITE trial, NICMP patients had a higher mortality rate than our group (14.1%). Of note, the follow-up time was twenty-nine ± fourteen months. However, these patients did not receive the optimal medical treatment and device therapy according to the current HF guidelines [20]. Another study showed a mortality rate of 12.1% in NICMP patients who received ACEI, ARB, or ARNI [21]. We revealed a better outcome in our study compared with other published studies, but our study has a limited follow-up time of one year. 

Ventricular tachyarrhythmias occur in patients suffering from HF; therefore, the impact of ARNI on these events was evaluated. In our study, ventricular arrhythmia involving VF, nsVT, and VT tended to be higher during the one-year follow-up in the ICMP group in contrast with the NICMP group, albeit without statistical significance (38.71% vs. 17.24%). However, the effect of ARNI compared with enalapril, irrespective of HF etiology in reducing sudden cardiac death and death from the deterioration of HF, is numerically superior [22]. Concerning this, Martens et al. provided information about the cardiac death mechanism, and they investigated the impact on the incidence of ventricular arrhythmias after switching from ACEI to ARNI. In a mean follow-up for one year, a reduction in VT/VF and nsVT events was observed (total episodes of VT/VF pre-*n* = 51 vs. post-*n* = 14, mean episodes of nsVT pre-*n* = 7.7 ± 11.8 vs. post-*n* = 3.7 ± 5.4). In the study of Martens et al., 69% of observed patients had ICMP and 31% of them NICMP [23]. In addition, De Diego et al. reported that ARNI reduced ventricular arrhythmias in HErEF patients, 82% from whom suffered ICMP [24]. Our data and published data indicate a possible impact of ARNI on arrhythmias. Further data to investigate this field are needed. 

Biochemical effects of the treatment with ARNI are presented in the NICMP group after therapy initiation, as reflected by circulating NT-proBNP (in ICMP, from 6266 to 6190 ng/L; in NICMP, from 5132 to 1170 ng/L). In this regard, the PIONEER-HF-trial presented that the treatment with ARNI in acutely decompensated patients was associated with a higher decrease in NT-proBNP than with the treatment with enalapril [25]. However, PIONEER-HF patients were not divided according to HF etiology. Consistent with the decrease in NT-proBNP, LVEF increased in our study after medication with ARNI regardless of HF etiology at one-year follow-up (in the ICMP group, from 24.13% ± 8.39 to 32.13% ± 7.53, and in the NICMP group, from 25.31% ± 7.68 to 29.63% ± 10.01). The improvement in LVEF after ARNI was also recently observed [26]. 

It is known that ARNI might worsen kidney function [27]. In our study, we observed that GFR decreased consistently with an increase in creatinine after the initiation of ARNI in ICMP patients. In NICMP, GFR increased consistently with a stable level of creatinine. In this regard, one study showed that creatinine serum increased slightly after the treatment with ARNI regardless of etiology [28]. In another study, an increase in serum creatinine and a decrease in GFR were observed, with a higher tendency in valsartan patients compared with ARNI patients [29]. In the present study, the increase in LVEF in NICMP patients could be the reason that kidney function improved in patients suffering from NICMP. On the other hand, other patients did not seem to have the ability to compensate due to the hemodynamic changes after the treatment with ARNI. 

In the multivariate analysis, the aldosterone antagonist was determined as a predictor for a reduction in mortality at one-year follow-up. A network meta-analysis reported that the current guideline recommendation in the treatment of HFrEF including ARNI, beta blocker, and aldosterone antagonist is superior compared with other all-medicated combinations to reduce all-cause mortality [30].

In summary, the etiology of HF plays an important role in choosing the best treatment for patients with chronic HF. ARNI seems to be effective in the treatment of HFrEF patients, with a tendency to improve cardiac function in ICMP patients and to decrease the mortality rate. However, ARNI might not impact the risk of ventricular tachyarrhythmias, particularly in ICMP patients. In addition, the improvement in renal function in NICMP patients compared with a depression of kidney function in ICMP patients is an interesting aspect. In this regard, the improvement in LVEF might lead to a lower rate of cardio-renal failure in patients suffering from NICMP.

## 5. Study Limitations

This study is a retrospective monocentric study. The number of patients suffering from ICMP and NICMP was relatively small when compared with other studies. In addition, bias due to unknown or unmeasured confounders cannot be excluded due to the retrospective nature of the study. LVEF was not systematically evaluated using, for example, cardiac magnetic resonance tomography. The NYHA class was assessed without using a qualitative evaluation questionnaire. Furthermore, some patients did not achieve the target dose in the ambulatory setting. We followed up with the patients only for twelve months. However, this study represents real-world clinical data that provide information about the effectiveness of ARNI in a heterogeneous population in clinical practice. The documentation of arrhythmias occurred by device interrogation.

## 6. Conclusions

Despite a small numerical decrease in the rates of malignant ventricular arrhythmias, the use of ARNI was not associated with differences in all-cause mortality in this small cohort of patients already treated with medical heart failure therapy and implanted devices. However, further studies are needed to investigate the impact of ARNI on the outcome according to HF etiology.

## Figures and Tables

**Table 1:** Baseline characteristics of NICMP and ICMP patients presenting at the beginning of the treatment with ARNI and one-year follow-up.

Variables	NICMP Patients before ARNI *n* = 60	ICMP Patients before ARNI *n* = 61	*p*-Value ^1^	NICMP Patients after ARNI *n* = 60	ICMP Patients after ARNI *n* = 61	*p*-Value ^2^
**Demographics**						
Age, mean ± SD	61.8 ± 11	69.7 ± 11	<0.001	-	-	**-**
Gender (male) *n*(%)	43/59 (72.9)	54/61 (88.5)	0.03	-	-	**-**
**Clinic parameter**						
Systolic BP mmHg, mean ± SD	128.73 ± 14.50	132.14 ± 30.28	0.28	113.81 ± 17.67	118.62 ± 27.17	0.23
Diastolic BP mmHg, mean ± SD	82.09 ± 9.27	76.43 ± 12.41	0.27	72.00 ± 12.10	69.52 ± 20.00	0.87
Heart rate Bpm, mean ± SD	76.30 ± 15.02	78.95 ± 16.61	0.48	71.34 ± 13.49	73.62 ± 18.86	0.72
**Laboratory values**						
GFR (ml/min), mean ± SD	60.63 ± 21.43	52.91 ± 26.02	0.002	70.52 ± 28.08	42.10 ±19.05	0.001
Creatinine (mg/dL), mean ± SD	1.19 ± 0.53	1.60 ± 0.52	0.01	1.19 ± 0.47	1.99 ± 1.24	0.02
Potassium (mmol/L), median (min.-max)	4.03 (2.1–5.10)	4.10 (3.29–5.70)	0.62	4.30 (3.10–6.60)	4.20 (3.40–6.20)	0.26
proBNP (ng/L), mean ± SD	5132.82 ± 6394.67	6266.68 ± 5794.73	0.24	1170.69 ± 1631.81	6190 ± 7623.41	0.01
**ECG Data mean ± SD**						
PQ-Time	170.80 ± 37.74	182.53 ± 29.91	0.29	164.46 ± 21.73	188.60 ± 43.72	0.08
QT-Time	427.11 ± 57.67	437.24 ± 66.05	0.59	422.85 ± 47.46	430.70 ± 40.31	0.25
QTc-time	470.14 ± 55.04	478.83 ± 44.39	0.45	455.92 ± 33.66	471.00 ± 39.30	0.35
**Medical history *n*(%)**						
Smoking	14/55 (25.45)	13/54 (24.07)	0.87	11/52 (21.15)	8/44(18.18)	0.72
Diabetes mellitus type II	16/58 (27.59)	24/61 (39.34)	0.18	18/59 (30.51)	24/58 (41.38)	0.22
Hypertension	34/54 (62.96)	46/60 (76.67)	0.11	36/55 (65.45)	40/52 (76.92)	0.19
COPD	10/59 (16.95)	7/61 (11.48)	0.39	13/60 (21.67)	8/53 (15.09)	0.37
Asthma	1/59 (1.64)	0/61 (0.00)	0.49	1/58 (0.00)	0/49 (0.00)	0.49
History of malignancy	5/59 (8.47)	6/60 (10.00)	1.00	4/57 (7.02)	5/53 (9.43)	0.74
Stroke	6/58 (10.34)	5/60 (8.33)	0.76	6/57 (10.53)	5/56 (8.93)	1.00
Bleeding	2/59 (3.39)	3/61 (4.92)	1.00	1/58 (1.72)	2/57 (3.51)	0.62
Atrial fibrillation	11/35 (31.4)	19/41 (46.3)	0.19	18/35 (51.4)	25/40 (62.5)	0.33
**NYHA-Classification**						
I	1/45 (2.22)	0/45 (0.00)	0.05	3/32 (9.38)	4/41 (9.76)	0.14
II	16/45 (35.56)	6/45 (13.33)	0.05	14/32 (43.75)	9/41 (21.95)	0.14
III	26/45 (57.78)	34/45 (75.56)	0.05	14/32 (43.75)	22/41 (53.66)	0.14
IV	2/45 (4.44)	5/45 (11.11)	0.05	1/32 (3.13)	6/41 (14.63)	0.14
EF (%), mean ± SD	25.31 ± 7.68	24.13 ± 8.39	0.58	29.63 ± 10.01	32.13 ± 7.53	0.25
**Electronic cardiac device *n*(%)**						
CRT	19/59 (32.20)	15/61 (24.59)	0.36	25/60 (41.67)	18/60 (30.00)	0.18
ICD	28/60 (46.67)	42/61 (68.85)	0.01	32/59 (54.24)	46/60 (76.67)	0.01
DDD	1/60 (1.67)	3/61 (4.91)	0.62	1/60 (1.67)	2/60 (3.33)	1.00
CCM	11/60 (18.33)	17/60 (28.33)	0.20	13/60 (21.67)	22/60 (36.67)	0.07
Vagus stimulation	1/60 (1.67)	0/59 (0.00)	1.00	1/58 (1.72)	0/59 (0.00)	0.50
**Drugs on admission *n*(%)**						
Beta-blocker	56/59 (94.92)	56/59 (94.92)	1.00	59/59 (100.00)	57/58 (98.28)	0.50
AT-II-Antagonist	14/58 (24.14)	19/60 (31.67)	0.36	-	-	-
Aldosterone antagonist	48/59 (81.36)	40/60 (66.67)	0.07	46/59 (77.97)	41/59 (69.49)	0.30
ACE-Inhibitor	36/58 (62.07)	30/59 (50.85)	0.22	0/57 (0.00)	0/56 (0.00)	-
**Antiarrhythmic drugs *n*(%)**						
Amiodarone	8/58 (13.79)	11/60 (18.33)	0.50	12/59 (20.34)	13/60 (21.67)	0.86

*p*-value ^1^ for the comparison between NICMP and ICMP before ARNI; *p*-value ^2^ for the comparison between NICMP and ICMP after ARNI; SD, standard deviation; ARNI, angiotensin receptor-neprilysin inhibitor; ICMP, ischemic cardiomyopathy; NICMP, non-ischemic cardiomyopathy; ECG, electrocardiogram; BP, blood pressure; GFR, glomerular filtration rate; pro-BNP, pro-B-type natriuretic peptide; COPD, chronic obstructive pulmonary disease; EF, ejection fraction; CRT, cardiac resynchronization therapy; ICD, implantable cardioverter-defibrillator; CCM, cardiac contractility modulation; AT-II-Antagonist, angiotensin II receptor antagonist; ACE, angiotensin-converting-enzyme.

**Table 2:** Tachyarrhythmia occurrence in ICMP and NICMP patients at baseline, 6-, and 12-month follow-ups.

Variables	Baseline and after ARNI *n* = 121	NICMP *n* = 60	ICMP *n* = 61	*p*-Value *
** Arrhythmia ** ***n*** **(%)**				
**Ventricular tachyarrhythmia**				
Baseline Ω	14/113 (12.39)	6/56 (10.71)	8/57 (14.04)	0.78
6 months	11/86 (12.79)	3/41 (7.32)	8/45 (17.78)	0.20
12 months	17/60 (28.33)	5/29 (17.24)	12/31 (38.71)	0.07
**Ventricular fibrillation**				
Baseline	3/113 (2.65)	2/56 (3.57)	1/57 (1.75)	0.62
6 months	3/86 (3.49)	0/41 (0.00)	3/45 (6.67)	0.24
12 months	5/60 (8.33)	1/29 (3.45)	4/31 (12.90)	0.36
**nsVT**				
Baseline	9/87 (10.34)	4/38 (10.53)	5/49 (10.20)	1.00
6 months	7/78 (8.97)	1/36 (2.78)	6/42 (14.29)	0.12
12 months	15/54 (27.78)	5/25 (20.00)	10/29 (34.48)	0.24
**Ventricular tachycardia**				
Baseline	5/113 (4.42)	3/56 (5.36)	2/57 (3.51)	0.68
6 months	4/86 (4.65)	2/41 (4.88)	2/45 (4.44)	1.00
12 months	8/60 (13.33)	3/29 (10.34)	5/31 (16.13)	0.71

* *p*-values for the comparison between NICMP and ICMP patients after ARNI; Ω, events during 6–12 months before ARNI; SD, standard deviation; nsVT, non-sustained ventricular tachycardia.

**Table 3:** Device therapy and the impact on the outcome.

**Device Type**	**CRT-D**	**ICD**	**DDD**	**CCM**	***p*** **-Value**
Clinical outcomes (%)					
Mortality	10	6.1	12.5	3.2	<0.001
Ventricular tachyarrhythmia	32	45.1	16.7	55.2	0.003
Ventricular fibrillation	20	18.3	16.7	20.6	<0.001
nsVT	20	32.4	0	34.5	<0.001
Ventricular tachycardia	20	26.8	0	27.6	<0.001

*p*-values for the comparison between device types; CRT, cardiac resynchronization therapy; ICD, implantable cardioverter-defibrillator; DDD, dual-chamber pacemaker; CCM, cardiac contractility modulation; nsVT, non-sustained ventricular tachycardia.

**Table 4:** Predictors for mortality.

Variable	*Univariate Analysis*			*Multivariate Analysis*		
	HR	95% CI	*p*-Value	HR	95% CI	*p*-Value
**Patients characteristic**						
Age > 65	7.43	0.95–58.02	0.06			
Gender	1.66	0.41–6.79	0.48			
NICMP	0.84	0.23–3.12	0.79			
ICMP	1.19	0.32–4.45	0.79			
**Medical History**						
Smoking	0.48	0.15–1.56	0.22			
DM type II	5.34	1.42–20.13	0.01	3.79	0.98–14.69	0.06
Hypertension	3.96	0.51–30.97	0.19			
COPD	1.03	0.22–4.79	0.97			
History of Malignancy	0.7	0.15–3.19	0.64			
Stroke	3.17	0.84–11.96	0.09			
Bleeding	2.29	0.29–17.89	0.43			
NYHA-Classification III and IV	31.98	0.02–43617.81	0.35			
**Electronic cardiac device**						
CRT-D	1.26	0.34–4.76	0.73			
ICD	0.39	0.12–1.31	0.13			
DDD	1.49	0.19–11.63	0.71			
CCM	0.27	0.04–2.13	0.22			
**Drugs on admission**						
Beta-blocker	21.39	0.00–129.5	0.65			
AT-II-Antagonist	1.56	0.46–5.33	0.48			
Aldosterone antagonist	0.16	0.04–0.59	0.006	0.21	0.05–0.82	0.03
ACE-Inhibitor	0.88	0.27–2.89	0.84			
Amiodarone	0.04	0.00–49.87	0.37			
** Arrhythmia before sacubitril/valsartan **						
Ventricular tachyarrhythmia	2.12	0.62–7.24	0.23			
Ventricular fibrillation	1.02	0.13–7.99	0.98			
nsVT	3.44	0.91–12.99	0.07			
Ventricular tachycardia	0.04	0.00–313.25	0.49			

Abbreviations: HR, hazard ratio; CI, confidence interval; DM, diabetes mellitus; COPD, chronic obstructive pulmonary disease; NYHA, New York Heart Association; CRT, cardiac resynchronization therapy; ICD, implantable cardioverter-defibrillator; DDD, dual-chamber pacemaker; CCM, cardiac contractility modulation; AT-II-Antagonist, angiotensin II receptor antagonist; ACE-Inhibitor, angiotensin-converting enzyme inhibitor; nsVT, non-sustained ventricular tachycardia.

## Data Availability

The data presented in this study are available on request from the corresponding author.
